# 4-Fluoro-*N*-[(*E*)-3,4,5-tri­meth­oxy­benzyl­idene]aniline

**DOI:** 10.1107/S1600536813017741

**Published:** 2013-07-10

**Authors:** R. K. Balachandar, S. Kalainathan, Shibu M. Eappen, Jiban Podder

**Affiliations:** a Centre for Crystal Growth, School of Advanced sciences, VIT University, Vellore 632 014, India; b Sophisticated Test and Instrumentation Centre (STIC), Cochin University PO, Cochin 682 022, Kerala, India; c Department of Physics, Bangladesh University of Engineering and Technology, Dhaka 1000, Bangladesh

## Abstract

The title compound, C_16_H_16_FNO_3_, exists in a *trans* configuration with respect to the C=N bond [1.258 (2) Å]. The central meth­oxy O atom deviates from the plane of the attached benzene ring by 0.0911 (14) Å. The dihedral angle between the aromatic rings is 47.58 (11)°. The crystal structure features C—H⋯N and C—H⋯O inter­actions.

## Related literature

For the uses and biological importance of Schiff base compounds, see: Xia *et al.* (2009 ▶); Shah *et al.* (1992 ▶); Ünver *et al.* (2004 ▶). For related structures, see: Fun *et al.* (2011 ▶); Khalaji & Simpson (2009 ▶); Balachandar *et al.* (2013 ▶). 
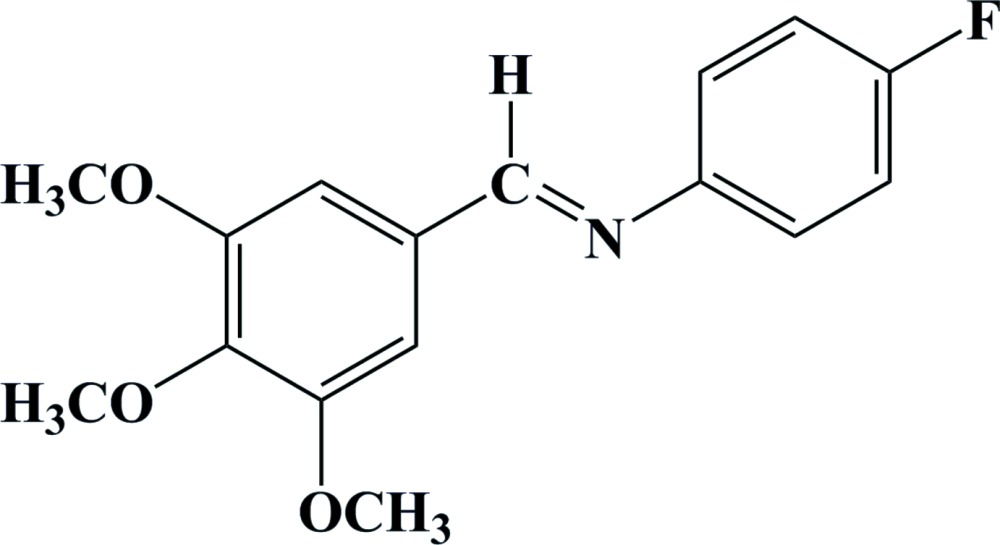


## Experimental

### 

#### Crystal data

C_16_H_16_FNO_3_
*M* *_r_* = 289.30
Monoclinic, 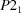

*a* = 7.1147 (9) Å
*b* = 8.3841 (9) Å
*c* = 12.9217 (13) Å
β = 105.266 (5)°
*V* = 743.59 (14) Å^3^
*Z* = 2
Mo *K*α radiation
μ = 0.10 mm^−1^
*T* = 296 K
0.40 × 0.35 × 0.30 mm

#### Data collection

Bruker Kappa APEXII CCD diffractometer
Absorption correction: multi-scan (*SADABS*; Bruker, 1999 ▶) *T*_min_ = 0.962, *T*_max_ = 0.971
5699 measured reflections
3358 independent reflections
2468 reflections with *I* > 2σ(*I*)
*R*_int_ = 0.017

#### Refinement

*R*[*F* ^2^ > 2σ(*F* ^2^)] = 0.040
*wR*(*F* ^2^) = 0.128
*S* = 1.00
3358 reflections
190 parameters
1 restraint
H-atom parameters constrained
Δρ_max_ = 0.14 e Å^−3^
Δρ_min_ = −0.13 e Å^−3^

### 

Data collection: *APEX2* (Bruker, 2004 ▶); cell refinement: *APEX2* and *SAINT* (Bruker, 2004 ▶); data reduction: *SAINT* and *XPREP* (Bruker, 2004 ▶); program(s) used to solve structure: *SIR92* (Altomare *et al.*, 1993 ▶); program(s) used to refine structure: *SHELXL97* (Sheldrick, 2008 ▶); molecular graphics: *ORTEP-3 for Windows* (Farrugia, 2012 ▶); software used to prepare material for publication: *SHELXL97* and *PLATON* (Spek, 2009 ▶).

## Supplementary Material

Crystal structure: contains datablock(s) global, I. DOI: 10.1107/S1600536813017741/ds2233sup1.cif

Structure factors: contains datablock(s) I. DOI: 10.1107/S1600536813017741/ds2233Isup2.hkl

Supplementary material file. DOI: 10.1107/S1600536813017741/ds2233Isup3.cml

Additional supplementary materials:  crystallographic information; 3D view; checkCIF report

## Figures and Tables

**Table 1:** Hydrogen-bond geometry (Å, °)

*D*—H⋯*A*	*D*—H	H⋯*A*	*D*⋯*A*	*D*—H⋯*A*
C4—H4⋯N1^i^	0.93	2.57	3.492 (3)	174
C7—H7⋯O3^ii^	0.93	2.58	3.504 (3)	173

Symmetry codes: (i) 
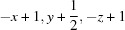
; (ii) 
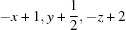
.

**Fig. 1.**
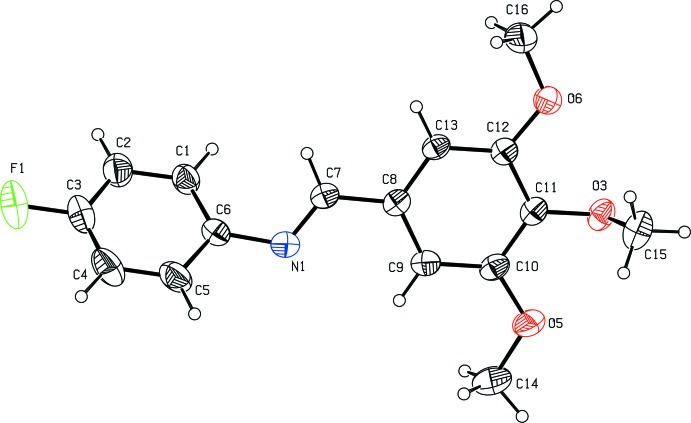
The molecular structure of the title compound with the atom numbering scheme, displacement ellipaoids are drawn at 30% probability level. H atoms are present as small spheres of arbitary radius.

**Fig. 2.**
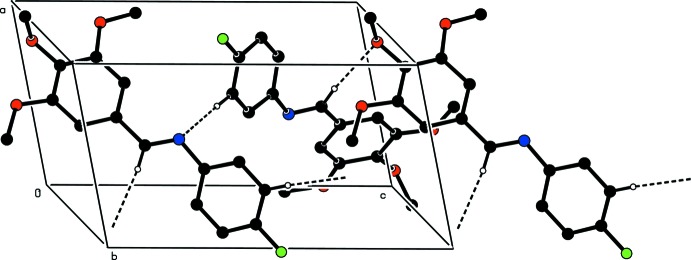
The crystal packing of the title compound, viewed down a-axis, showing C4—H4···N1i and C7—H7···O3ii inter-molecular interactions. The H atoms not involved in the bonding have been excluded for clarity.

## supplementary crystallographic information

### Comment

Schiff bases are among the most useful ligands in coordination chemistry as they readily form stable complexes with most transition metals (Xia *et al.*, 2009). They are known to exibit potent anti-bacterial, anti-convulsant, anti-inflammatory and anti-cancer activities (Shah *et al.*, 1992). In addition to that, it shows the Non-linear optical properties (Ünver *et al.*, 2004). Therefore, successful application of Schiff bases requires a careful study of their characteristics.

The title compound, C_16_ H_16_ F N O_3_, exists in a *trans* configuration with respect to the C═N bond [1.258 (2) Å]. The N1═C8 bond length of 1.258 (2) Å is shorter than the N–C bond [1.411 (3) Å], indicating a typical imine double bond. Moreover, the C–N–C angle is 118.93 (17) °. X-ray analysis confirms the molecular structure and atom connectivity as illustrated in Fig. 1.

The dihedral angle formed between the phenyl rings (C1–C6) and (C8–C13) is 47.58 (11) °. The flurine atom F1 is deviated from the phenyl ring (C1–C6) by -0.0243 (23) Å. One of the oxygen atom attached to the phenyl ring (C8–C13) is deviated from the same plane by -0.0911 (14) Å.

The crystal packing is stabilized by C4—H4···N1^i^ and C7—H7···O3^ii^ inter-molecular interactions. The symmetry codes: (i). 1 - *x*,1/2 + *y*,1 - *z*. (ii). 1 - *x*,1/2 + *y*,2 - *z*.

### Experimental

The title compound was prepared by the condensation reaction between of 3,4,5-trimethoxybenzaldehyde (1 mmol, 0.196 g) and 4-Fluoroaniline (1 mmol, 0.172 g), which were taken in equimolar ratio and dissolved in methanol (20 ml). The resulting mixture was stirred at room temperature for overnight. Then filtering, drying the synthesized compound dissolved in a 20 ml of methanol to purify the title material by recrystallization process at least for three times. After 4 days, colourless single crystals were obtained using methanol as solvent by keeping the solution for slow evaporation suitable for single-crystal X-ray structure analysis.

### Refinement

The positions of hydrogen atoms were localized from the difference electron density maps and their distances were geometrically constrained. The H atoms bound to the C atoms were treated as riding atoms, with d(C–H) = 0.93 Å and *U*_iso_(H) = 1.2*U*_eq_(C) for aryl atoms. d(C–H) = 0.96 Å and *U*_iso_(H) = 1.5*U*_eq_(C) for methyl group. The rotation angles for methyl group is optimized by least squares. During the diffraction experiment, 998 Friedel pairs were merged.

### Crystal data

**Table:**

C_16_H_16_FNO_3_	*F*(000) = 304
*M**_r_* = 289.30	*D*_x_ = 1.292 Mg m^−^^3^
Monoclinic, *P*2_1_	Mo *K*α radiation, λ = 0.71073 Å
Hall symbol: P 2yb	Cell parameters from 2468 reflections
*a* = 7.1147 (9) Å	θ = 2.9–28.3°
*b* = 8.3841 (9) Å	µ = 0.10 mm^−^^1^
*c* = 12.9217 (13) Å	*T* = 296 K
β = 105.266 (5)°	Block, colourless
*V* = 743.59 (14) Å^3^	0.40 × 0.35 × 0.30 mm
*Z* = 2	

### Data collection

**Table:**

Bruker Kappa APEXII CCD diffractometer	3358 independent reflections
Radiation source: fine-focus sealed tube	2468 reflections with *I* > 2σ(*I*)
Graphite monochromator	*R*_int_ = 0.017
ω and φ scan	θ_max_ = 28.3°, θ_min_ = 2.9°
Absorption correction: multi-scan (*SADABS*; Bruker, 1999)	*h* = −6→9
*T*_min_ = 0.962, *T*_max_ = 0.971	*k* = −11→11
5699 measured reflections	*l* = −17→16

### Refinement

**Table:**

Refinement on *F*^2^	Primary atom site location: structure-invariant direct methods
Least-squares matrix: full	Secondary atom site location: difference Fourier map
*R*[*F*^2^ > 2σ(*F*^2^)] = 0.040	Hydrogen site location: inferred from neighbouring sites
*wR*(*F*^2^) = 0.128	H-atom parameters constrained
*S* = 1.00	*w* = 1/[σ^2^(*F*_o_^2^) + (0.0767*P*)^2^ + 0.017*P*] where *P* = (*F*_o_^2^ + 2*F*_c_^2^)/3
3358 reflections	(Δ/σ)_max_ < 0.001
190 parameters	Δρ_max_ = 0.14 e Å^−^^3^
1 restraint	Δρ_min_ = −0.13 e Å^−^^3^

### Special details

**Table:**

Geometry. All e.s.d.'s (except the e.s.d. in the dihedral angle between two l.s. planes) are estimated using the full covariance matrix. The cell e.s.d.'s are taken into account individually in the estimation of e.s.d.'s in distances, angles and torsion angles; correlations between e.s.d.'s in cell parameters are only used when they are defined by crystal symmetry. An approximate (isotropic) treatment of cell e.s.d.'s is used for estimating e.s.d.'s involving l.s. planes.
Refinement. Refinement of *F*^2^ against ALL reflections. The weighted *R*-factor *wR* and goodness of fit *S* are based on *F*^2^, conventional *R*-factors *R* are based on *F*, with *F* set to zero for negative *F*^2^. The threshold expression of *F*^2^ > σ(*F*^2^) is used only for calculating *R*-factors(gt) *etc*. and is not relevant to the choice of reflections for refinement. *R*-factors based on *F*^2^ are statistically about twice as large as those based on *F*, and *R*- factors based on ALL data will be even larger.

### Fractional atomic coordinates and isotropic or equivalent isotropic displacement parameters (Å^2^)

**Table:**

	*x*	*y*	*z*	*U*_iso_*/*U*_eq_	
F1	0.9942 (4)	0.5751 (3)	0.51013 (17)	0.1191 (7)	
N1	0.4847 (3)	0.2770 (2)	0.70169 (14)	0.0557 (4)	
O3	0.0659 (2)	−0.05790 (17)	1.02505 (12)	0.0596 (4)	
C4	0.6764 (5)	0.5185 (4)	0.5155 (2)	0.0901 (9)	
H4	0.6295	0.5810	0.4548	0.108*	
O5	−0.0273 (2)	−0.0800 (2)	0.81378 (13)	0.0745 (5)	
O6	0.3531 (2)	0.12728 (18)	1.13224 (11)	0.0608 (4)	
C3	0.8696 (5)	0.5003 (3)	0.5578 (2)	0.0763 (7)	
C2	0.9455 (4)	0.4108 (4)	0.6458 (2)	0.0737 (7)	
H2	1.0795	0.3987	0.6724	0.088*	
C6	0.6199 (3)	0.3532 (3)	0.65551 (15)	0.0530 (5)	
C7	0.5225 (3)	0.2666 (2)	0.80220 (16)	0.0474 (4)	
H7	0.6363	0.3137	0.8430	0.057*	
C8	0.3977 (3)	0.1847 (2)	0.85798 (15)	0.0453 (4)	
C13	0.4394 (3)	0.1984 (2)	0.96847 (15)	0.0465 (4)	
H13	0.5447	0.2598	1.0053	0.056*	
C11	0.1708 (3)	0.0279 (2)	0.96978 (16)	0.0482 (4)	
C1	0.8184 (3)	0.3381 (3)	0.69503 (18)	0.0605 (6)	
H1	0.8679	0.2774	0.7564	0.073*	
C12	0.3258 (3)	0.1214 (2)	1.02398 (15)	0.0468 (4)	
C15	−0.0943 (4)	0.0258 (3)	1.0437 (2)	0.0794 (7)	
H15A	−0.1612	−0.0411	1.0827	0.119*	
H15B	−0.1819	0.0552	0.9763	0.119*	
H15C	−0.0488	0.1201	1.0847	0.119*	
C5	0.5502 (4)	0.4435 (4)	0.56337 (18)	0.0777 (7)	
H5	0.4166	0.4532	0.5338	0.093*	
C9	0.2415 (3)	0.0927 (2)	0.80236 (17)	0.0513 (5)	
H9	0.2132	0.0838	0.7281	0.062*	
C10	0.1283 (3)	0.0142 (2)	0.85863 (16)	0.0513 (5)	
C14	−0.0696 (4)	−0.1114 (4)	0.7021 (2)	0.0955 (10)	
H14A	−0.1824	−0.1788	0.6812	0.143*	
H14B	0.0396	−0.1639	0.6864	0.143*	
H14C	−0.0948	−0.0128	0.6632	0.143*	
C16	0.5085 (4)	0.2205 (4)	1.1925 (2)	0.0863 (8)	
H16A	0.5116	0.2145	1.2671	0.129*	
H16B	0.4905	0.3294	1.1691	0.129*	
H16C	0.6291	0.1811	1.1824	0.129*	

### Atomic displacement parameters (Å^2^)

**Table:**

	*U*^11^	*U*^22^	*U*^33^	*U*^12^	*U*^13^	*U*^23^
F1	0.1440 (16)	0.1259 (17)	0.1116 (12)	−0.0088 (14)	0.0766 (12)	0.0330 (12)
N1	0.0526 (10)	0.0608 (10)	0.0501 (9)	−0.0022 (8)	0.0073 (7)	−0.0009 (8)
O3	0.0550 (8)	0.0458 (8)	0.0784 (10)	−0.0049 (6)	0.0179 (7)	0.0129 (7)
C4	0.115 (3)	0.096 (2)	0.0619 (14)	0.0216 (18)	0.0283 (15)	0.0319 (15)
O5	0.0697 (10)	0.0754 (11)	0.0697 (9)	−0.0290 (9)	0.0030 (8)	−0.0048 (9)
O6	0.0667 (9)	0.0585 (9)	0.0548 (8)	−0.0136 (7)	0.0117 (7)	0.0029 (7)
C3	0.103 (2)	0.0727 (16)	0.0654 (15)	0.0007 (14)	0.0434 (15)	0.0111 (12)
C2	0.0710 (15)	0.0847 (18)	0.0699 (14)	0.0018 (13)	0.0266 (12)	0.0071 (13)
C6	0.0614 (13)	0.0531 (11)	0.0437 (10)	0.0027 (9)	0.0125 (9)	−0.0014 (9)
C7	0.0448 (10)	0.0406 (10)	0.0540 (11)	0.0010 (8)	0.0077 (8)	−0.0028 (8)
C8	0.0416 (9)	0.0364 (9)	0.0562 (11)	0.0053 (7)	0.0097 (8)	0.0019 (8)
C13	0.0425 (9)	0.0379 (9)	0.0558 (11)	−0.0014 (7)	0.0071 (8)	−0.0019 (8)
C11	0.0445 (10)	0.0345 (9)	0.0638 (11)	0.0031 (8)	0.0111 (9)	0.0074 (8)
C1	0.0630 (14)	0.0647 (13)	0.0543 (12)	0.0057 (10)	0.0164 (10)	0.0139 (10)
C12	0.0481 (10)	0.0363 (9)	0.0546 (11)	0.0033 (8)	0.0110 (8)	0.0039 (8)
C15	0.0658 (15)	0.0681 (16)	0.113 (2)	−0.0018 (12)	0.0385 (14)	0.0099 (14)
C5	0.0791 (15)	0.099 (2)	0.0506 (12)	0.0123 (15)	0.0093 (11)	0.0191 (13)
C9	0.0511 (10)	0.0456 (10)	0.0522 (10)	0.0007 (8)	0.0047 (8)	−0.0010 (8)
C10	0.0454 (10)	0.0378 (10)	0.0650 (12)	−0.0016 (8)	0.0049 (9)	0.0012 (9)
C14	0.0864 (19)	0.102 (2)	0.0858 (18)	−0.0330 (17)	0.0016 (16)	−0.0256 (17)
C16	0.097 (2)	0.097 (2)	0.0620 (14)	−0.0355 (17)	0.0170 (14)	−0.0132 (14)

### Geometric parameters (Å, º)

**Table:**

F1—C3	1.359 (3)	C8—C13	1.384 (3)
N1—C7	1.258 (2)	C8—C9	1.388 (3)
N1—C6	1.411 (3)	C13—C12	1.375 (3)
O3—C11	1.366 (2)	C13—H13	0.9300
O3—C15	1.412 (3)	C11—C12	1.384 (2)
C4—C3	1.347 (4)	C11—C10	1.392 (3)
C4—C5	1.370 (4)	C1—H1	0.9300
C4—H4	0.9300	C15—H15A	0.9600
O5—C10	1.359 (2)	C15—H15B	0.9600
O5—C14	1.419 (3)	C15—H15C	0.9600
O6—C12	1.362 (2)	C5—H5	0.9300
O6—C16	1.409 (3)	C9—C10	1.386 (3)
C3—C2	1.351 (4)	C9—H9	0.9300
C2—C1	1.378 (3)	C14—H14A	0.9600
C2—H2	0.9300	C14—H14B	0.9600
C6—C1	1.375 (3)	C14—H14C	0.9600
C6—C5	1.388 (3)	C16—H16A	0.9600
C7—C8	1.455 (3)	C16—H16B	0.9600
C7—H7	0.9300	C16—H16C	0.9600
			
C7—N1—C6	118.93 (17)	O6—C12—C13	125.09 (17)
C11—O3—C15	113.85 (16)	O6—C12—C11	114.78 (16)
C3—C4—C5	119.0 (2)	C13—C12—C11	120.13 (17)
C3—C4—H4	120.5	O3—C15—H15A	109.5
C5—C4—H4	120.5	O3—C15—H15B	109.5
C10—O5—C14	118.18 (19)	H15A—C15—H15B	109.5
C12—O6—C16	117.81 (17)	O3—C15—H15C	109.5
C4—C3—C2	122.9 (3)	H15A—C15—H15C	109.5
C4—C3—F1	118.8 (2)	H15B—C15—H15C	109.5
C2—C3—F1	118.3 (3)	C4—C5—C6	120.6 (3)
C3—C2—C1	117.9 (2)	C4—C5—H5	119.7
C3—C2—H2	121.0	C6—C5—H5	119.7
C1—C2—H2	121.0	C10—C9—C8	119.28 (18)
C1—C6—C5	117.9 (2)	C10—C9—H9	120.4
C1—C6—N1	123.28 (19)	C8—C9—H9	120.4
C5—C6—N1	118.7 (2)	O5—C10—C9	124.98 (18)
N1—C7—C8	123.50 (17)	O5—C10—C11	114.75 (18)
N1—C7—H7	118.3	C9—C10—C11	120.27 (17)
C8—C7—H7	118.3	O5—C14—H14A	109.5
C13—C8—C9	120.36 (17)	O5—C14—H14B	109.5
C13—C8—C7	118.60 (16)	H14A—C14—H14B	109.5
C9—C8—C7	121.03 (17)	O5—C14—H14C	109.5
C12—C13—C8	120.21 (16)	H14A—C14—H14C	109.5
C12—C13—H13	119.9	H14B—C14—H14C	109.5
C8—C13—H13	119.9	O6—C16—H16A	109.5
O3—C11—C12	120.42 (17)	O6—C16—H16B	109.5
O3—C11—C10	119.75 (17)	H16A—C16—H16B	109.5
C12—C11—C10	119.73 (17)	O6—C16—H16C	109.5
C6—C1—C2	121.5 (2)	H16A—C16—H16C	109.5
C6—C1—H1	119.2	H16B—C16—H16C	109.5
C2—C1—H1	119.2		

### Hydrogen-bond geometry (Å, º)

**Table:**

*D*—H···*A*	*D*—H	H···*A*	*D*···*A*	*D*—H···*A*
C4—H4···N1^i^	0.93	2.57	3.492 (3)	174
C7—H7···O3^ii^	0.93	2.58	3.504 (3)	173

Symmetry codes: (i) −*x*+1, *y*+1/2, −*z*+1; (ii) −*x*+1, *y*+1/2, −*z*+2.
